# Understanding Determinants of Health Care Professionals’ Perspectives on Mobile Health Continuance and Performance

**DOI:** 10.2196/12350

**Published:** 2019-03-18

**Authors:** Ju-Ling Hsiao, Rai-Fu Chen

**Affiliations:** 1 Department of Hospital and Health Care Administration Chia-Nan University of Pharmacy and Science Tainan City Taiwan; 2 Department of Information Management Chia-Nan University of Pharmacy and Science Tainan City Taiwan

**Keywords:** delivery of health care, mobile health, health information management, health care quality, access, and evaluation

## Abstract

**Background:**

With the widespread use of mobile technologies, mobile information systems have become crucial tools in health care operations. Although the appropriate use of mobile health (mHealth) may result in major advances in expanding health care coverage (increasing decision-making speeds, managing chronic conditions, and providing suitable health care in emergencies), previous studies have argued that current mHealth research does not adequately evaluate mHealth interventions, and it does not provide sufficient evidence regarding the effects on health.

**Objective:**

The aim of this study was to facilitate the widespread use of mHealth systems; an accurate evaluation of the systems from the users’ perspective is essential after the implementation and use of the system in daily health care practices. This study extends the expectation-confirmation model by using characteristics of individuals, technology, and tasks to identify critical factors affecting mHealth continuance and performance from the perspective of health care professionals (HCPs).

**Methods:**

A questionnaire survey was used to collect data from HCPs who were experienced in using mHealth systems of a Taiwanese teaching hospital. In total, 282 questionnaires were distributed, and 201 complete and valid questionnaires were returned, thus indicating a valid response rate of 71.3% (201/282). The collected data were analyzed using WarpPLS version 5.0 (ScriptWarp Systems).

**Results:**

The results revealed that mHealth continuance (*R*^2^=0.522) was mainly affected by perceived usefulness, technology maturity, individual habits, task mobility, and user satisfaction, whereas individual performance (*R*^2^=0.492) was affected by mHealth continuance. In addition, user satisfaction (*R*^2^=0.548) was affected by confirmation and perceived usefulness of mHealth, whereas perceived usefulness (*R*^2^=0.521) was affected by confirmation. This implied that confirmation played a key role in affecting perceived usefulness and user satisfaction. Furthermore, the results showed that mHealth continuance positively affected individual performance.

**Conclusions:**

The identified critical factors influencing mHealth continuance and performance can be used as a useful assessment tool by hospitals that have implemented mHealth systems to facilitate the use and infusion of the systems. Furthermore, the results can help health care institutions that intend to introduce or develop mHealth applications to identify critical issues and effectively allocate limited resources to mHealth systems.

## Introduction

### Background

With the widespread use of mobile technologies, mobile information systems (ISs) have become crucial tools in health care operations. In recent times, smart health (sHealth) has become a critical strategy that is promoted by the government and medical industry; however, the successful implementation of sHealth depends on the development of mobile health (mHealth) [1]. mHealth is defined as health care to anyone, anytime, and anywhere by removing location and temporal constraints while improving both the coverage and quality of health care [2].

### Research Motivations and Purpose

In reality, health care professionals (HCPs) often require high-quality communication and information resources, including communication capabilities, hospital information systems (HISs), information resources, and clinical software applications, at the point of care to facilitate rapid decision making with a low error rate, improve the quality of data management and accessibility, and improve practice efficiency and knowledge [3-8]. Although the appropriate use of mHealth may result in major advances in expanding health care coverage, increasing decision-making speeds, managing chronic conditions, and providing suitable health care in emergencies [9], Solanas et al [1] argued that mHealth is still in its early stages of development. Mechael et al [10] reported that current mHealth research does not adequately evaluate mHealth interventions, and it does not provide sufficient evidence regarding the effects on health. In addition, the World Health Organization [11] indicated that competing priorities, cost, and lack of knowledge are the most crucial barriers to mHealth implementation. Thus, appropriate evaluations, specifically after the implementation of mHealth systems and their use in daily health care practice, are critical, particularly from users’ (HCPs’) perspectives. This paper proposes and validates an extended model by integrating the expectation-confirmation model (ECM) and the characteristics of individuals, technology, and tasks to identify critical factors affecting mHealth continuance and performance from the perspective of HCPs and assessing the infusion of mHealth in clinical practice.

### Literature Review

#### Mobile Health

Varshney [2] defined mHealth as health care to anyone, anytime, and anywhere by removing locational and temporal constraints while improving both the coverage and quality of health care. Alternatively, mHealth is the application of mobile communication technology in the field of health care; it integrates HISs and mobile devices with wireless communication technologies to achieve immediate medical care and handle diverse cooperative medical tasks [12]. Nowadays, various mobile devices—personal digital assistants, tablet personal computers (PCs), notebook computers, personal handy-phone system, smartphones, panel PCs, mobile clinical assistants, and iPads—have been used in accessing mobile ISs through wireless networks in clinical settings owing to their portable size, relatively low costs, and ease of use [13,14]. The term *mobile* emphasizes various abilities and conditions as well as movability and portability. Increasing mobility can also enhance service efficiency and flexibility. Previous studies have indicated that the need for mobility is the primary reason for the applications of technological innovations in hospitals, and mobility is crucial in health care [14-16]. Thus, mHealth has the potential to increase the speed, work quality, and efficiency of HCPs. The implementation of mHealth is often achieved using portable information devices, such as a tablet PC, notebook, iPad, or smartphone, to appropriately address the needs of HCPs.

Many studies have reported that when appropriately used, mHealth systems facilitate rapid decision making with low error rates, thereby improving the quality of data management and accessibility and improving practice efficiency and knowledge [3-8]. Some researchers [17-19] mentioned that mHealth systems improve the quality of health care services, increase the productivity of HCPs, and ensure the timeliness of information provision, thus reducing the occurrence of errors. Therefore, mHealth systems are expected to exert considerable effects on clinical routines and workflows.

#### Information Technology Continuance and Performance

Bhattacherjee [20] argued that existing information technology (IT) or information system (IS) acceptance models, focusing on user evaluations at the early stage of IT or IS adoption and implementation, provide an inadequate explanation of and may sometimes contradict observed continuance behaviors; moreover, the long-term success of an IT or IS depends on its continued use rather than its first-time use. Bhattacherjee [20] proposed an ECM, one of the earliest IS continuance models, based on expectation-confirmation theory [21] in consumer behavior for understanding IS continuance after implementation, where the use of ISs transcends conscious behavior and becomes part of the normal routine activity. The study revealed that users’ willingness to continue using ISs was affected by user satisfaction and perceived usefulness after using ISs. Moreover, the expectation-confirmation and perceived usefulness of ISs directly affect IS users’ satisfaction; user satisfaction directly affects the willingness to continue using ISs. Limayem et al [22] further suggested that information communication technology (ICT) implementation should be considered a success when a significant number of users progress from the initial adoption stage to using ICT on a continuing basis. Nowadays, the ECM is being widely used and extended to investigate factors affecting user intentions regarding ISs after IS implementation and behaviors in various research contexts, including Web portals [23], online communities [24], electronic medical records [25], mHealth systems [26], and e-service [27-30]. Among the aforementioned studies, Akter et al [26] considered that continuance is a challenge for mHealth systems and that exploring theories on continuance behavior is necessary for developing a comprehensive continuance model for understanding mHealth services. Thus, Akter et al [26] incorporated the ECM and the constructs of service quality and trust to investigate the continuance of mHealth services at the bottom of the economic pyramid. Mettler [25] integrated the ECM and factors affecting automatic behavior (facilitating conditions, task fit, and computer literacy) to evaluate electronic medical record continuance behavior. Furthermore, Chen et al [29] investigated the effects of technology readiness (innovativeness, optimism, discomfort, and insecurity) on user satisfaction and continued intention of e-services. Although many extended ECM studies were conducted, Bhattacherjee and Barfar [31] argued that some studies are inappropriate to just integrate acceptance and continuance theory to predict IS continuance behavior. This implied that the extended ECM should consider some salient variables in the IS Infusion (assimilation or integration) stage that a specific IS has been well implemented and become a part of their daily routine processes.

Some studies have emphasized examining the determinants of mHealth in the assimilation or integration stage, where the mHealth services or systems are stable and have been incorporated into routine practices [17,32-34]. For example, O’Connor et al [33] argued that most infusion studies have paid considerable attention to the technological aspects at an organization level rather than at an individual level. The authors suggested that additional studies be conducted at the technology infusion stage at an individual level by considering characteristics of technology, individuals, and tasks. Therefore, they proposed a research framework that focused on investigating the effects of the characteristics of technology (availability, maturity, and portability), individuals (habits, self-efficacy, and technology trust), and tasks (time criticality, interdependence, and mobility) on the extent of the infusion of mHealth services by HCPs and the relationship between the extent of infusion (including integrative use and exploratory use) and performance based on the results of an in-depth case study. Although the study proposed many potential factors influencing mHealth systems in the infusion stage, the framework should be appropriately modified and validated according to various health care contexts or applications.

The performance of mHealth ISs should be evaluated based on user satisfaction and the specific outcomes of their continued use from users’ perspectives as performance evaluation is a major concern of the effects of ITs or ISs [35-37]. Goodhue and Thompson [37] proposed the task-technology fit (TTF) theory to highlight the importance of the fit between the characteristics of technologies and user tasks in achieving the effects of individual performance. In the model, the TTF is affected by antecedents (including technologies and tasks characteristics), and the TTF also has a significant effect on IS utilization and performance. In addition, IS utilization also has a direct effect on individual performance in TTF theory. On the basis of the TTF perspective, Hsiao and Chen [32] found that the use of mobile ISs provided nursing staff with real-time and accurate information and increased their efficiency and effectiveness in patient-care duties, thus further improving nursing performance. Lin [17] reported a significant effect of the fit among technology (applicability, user interface, and portability), individual (computer self-efficacy, user experience, and self-immersion), and task (nonroutine, timeliness, interdependence, and mobility) characteristics on task performance (in terms of meeting expectations, positive attitude, and meeting user needs) of mHealth systems. Although the TTF theory provides a useful perspective for investigating the relationships among TTF, utilization, and performance, there is a lack of empirical studies, particularly in the health care industry in exploring the role of TTF in the IS infusion stage about IS being integrative and exploratory used [38]. In this study, we only refer to the results of antecedents of TTF and the relationship between IS utilization and individual performance of health information technologies’ applications.

## Methods

### Research Model

To provide comprehensive understanding and insights into the postimplementation stage of mHealth systems (or at IS infusion stage), this study proposed an extended ECM research model for investigating key factors affecting the continuance and performance of mHealth services in Taiwan by incorporating the ECM proposed by Bhattacherjee [20] and the framework of mHealth infusion proposed by O’Connor et al [33]. This integration is based on the assumption that mHealth continuance is critical in the IS infusion stage because the use of mHealth services has become a part of daily clinical practice for HCPs. Moreover, the continuance intentions of HCPs and the subsequent use behavior of mHealth systems are expected to enhance individual performance. However, some variables mentioned in the framework proposed by O’Connor et al [33] should be adjusted according to the health care contexts and applications in Taiwan. In this study, the variables of self-efficacy and technology trust of user characteristics in O’Connor et al [33] were excluded. Self-efficacy is not considered to be a significant factor in mHealth infusion because it is insignificant in studies of physicians’ [39] and nurses’ [40] HIS acceptance in Taiwan. Furthermore, the technology trust proposed by O’Connor et al [33] to address the problem that users may be reluctant to use the IT because of its reliability. In this study, the mHealth applications have been used in the case hospital for several years, and they have been infused into HCPs’ daily clinical practices. In addition, mHealth applications are not mandatorily used by HCPs; therefore, the technology trust is not a major concern in this study. We also append personal innovativeness as an investigated factor in individual characteristics as Rai et al reported that consumers’ personal innovativeness exerted significantly positive effects on mHealth usage intention and assimilation [34]. However, the relationship between personal innovativeness and mHealth continuance should be further validated in the health care contexts of Taiwan.

**Figure 1:**
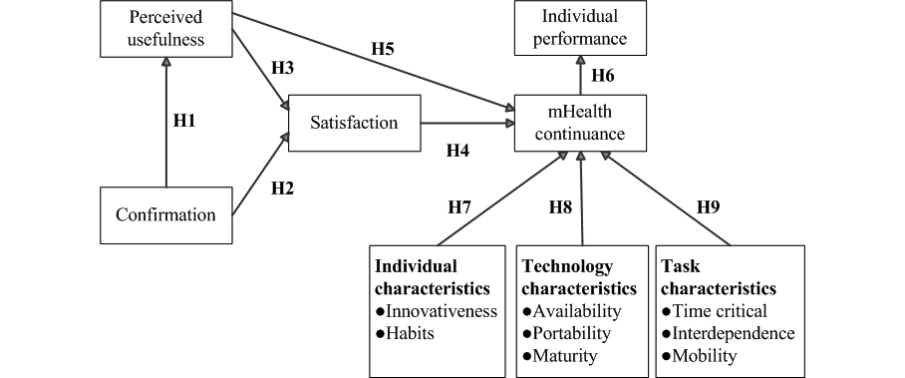
Research framework. H1: The confirmation of mHealth systems significantly affects perceived usefulness; H2: The confirmation of mHealth systems significantly affects user satisfaction; H3: The perceived usefulness of mHealth systems significantly affects user satisfaction; H4: User satisfaction with mHealth systems significantly affects mHealth continuance; H5: The perceived usefulness of mHealth systems significantly affects mHealth continuance; H6: The continuance of mHealth significantly affects individual performance; H7: The individual characteristics of HCPs significantly affect mHealth continuance; H8: The technology characteristics of mHealth significantly affect mHealth continuance; H9: The task characteristics of HCPs significantly affect mHealth continuance; mHealth: mobile health.

Therefore, the research model (Figure 1) can be divided into 2 major parts. The first part includes the aspects affecting electronic health (eHealth) continuance and performance derived from the ECM and the effects of mHealth continuance: confirmation, perceived usefulness, user satisfaction, continuance intention, and individual performance.

**Table 1:** Measurement and operational definitions of variables.

Construct		Operational definition	Source	Measurement items
Confirmation		Users’ perception of the congruence between expectation of mHealth^a^ use and its actual performance	[20]	4
Perceived usefulness		Users’ perception of the expected benefits of mHealth use	[21,41]	5
User satisfaction		Users’ affect with (feelings about) mHealth use	[20,42]	3
mHealth continuance		Users’ intention to continue using mHealth	[20,25]	3
**Individual**				
	Habits	The extent to which an individual tends to use the mHealth automatically	[22,33]	4
	Innovativeness	Willingness to try out any new technology	[29,41]	4
**Technology**				
	Availability	The ability of accessing patient information when required	[17,33]	3
	Portability	The degree of ease associated with transporting the mHealth	[17,33]	3
	Maturity	The existence of a level of system quality that is perceived as satisfactory and the perceived need for system improvement by the user.	[33,43]	3
**Task**				
	Time critical	The urgency when accessing information through the mHealth	[17,33]	3
	Interdependence	The degree to which completing tasks using mHealth requires interaction with other people	[17,32,33]	3
	Mobility	The extent to which a task is being performed in different locations using the mHealth	[17,33]	3
Individual performance		The use of mHealth can help health care practitioner improve efficiency, effectiveness, and quality of medical activities	[33,44]	6

^a^mHealth: mobile health.

The second part investigates the effect of characteristics of technology, individual, and task on mHealth continuance; this part is based on the framework proposed by O’Connor et al [33], which involves innovativeness and habits (individuals), availability, portability, maturity (technology), time criticality, interdependence, and mobility (tasks). The measurement, operational definition, and the number of items for the variables are summarized in Table 1.

In this study, confirmation refers to the users’ perception of the congruence between expectation associated with the use of mHealth systems and their actual performance. Perceived usefulness refers to the users’ perception of the expected benefits of mHealth use. User satisfaction refers to users’ affect (feelings) regarding previous mHealth use. As shown in the ECM, confirmation has a direct effect on perceived usefulness and user satisfaction [20]. Previous studies have argued that perceived usefulness directly affects user satisfaction and continuance of the use of system [20,25], and user satisfaction exerts direct effects on the continuance of the use of ISs [20,25,42]. Furthermore, mHealth continuance refers to the users’ intention to continue using mHealth. Individual performance refers to the use of mHealth to help HCPs improve efficiency, effectiveness, and quality of medical activities. Individual performance implies an increase in efficiency and improvement in work efficacy and quality [37,44]. O'Connor et al [33] argued that users’ continuance intentions could be affected by user satisfaction, and it can be used to predict or explain the continued use of IS as well as individual performance. Thus, 6 research hypotheses are proposed as follows:

- H1: The confirmation of mHealth systems significantly affects perceived usefulness.
- H2: The confirmation of mHealth systems significantly affects user satisfaction.
- H3: The perceived usefulness of mHealth systems significantly affects user satisfaction.
- H4: User satisfaction with mHealth systems significantly affects mHealth continuance.
- H5: The perceived usefulness of mHealth systems significantly affects mHealth continuance.
- H6: The continuance of mHealth significantly affects individual performance.

Most factors mentioned in the framework proposed by O’Connor et al [33] are suitable for mHealth continuance as continuance is critical in the mHealth infusion stage in which the use of mHealth services has become a part of daily clinical practice routine of HCPs. The influencing factors investigated in this study can be divided into 3 dimensions: individual characteristics (innovativeness and habits), technology characteristics (availability, maturity, and portability), and task characteristics (time criticality, interdependence, and mobility). The aforementioned individual characteristics represent individual traits and perceptions after using IT. Personal innovativeness is defined as a personal willingness to try using new information technologies [45], and it is a personal trait in technology-usage behavior [15,46]. Users with high innovativeness are often concerned with the development of new technologies, effectively learning technology functions on their own and providing suggestions to others. Rai et al [34] reported that consumers’ personal innovativeness exerted significantly positive effects on mHealth usage intention and assimilation. Thus, high personal innovativeness facilitates changes in organizations and the diffusion of implemented technologies in internal operations [45]. Chen et al [29] reported that innovativeness is a major driver of user satisfaction, and users with higher innovativeness exhibit a relatively high level of satisfaction with technologies and the willingness to continue using them. Therefore, personal innovativeness significantly affects mHealth continuance. Habit has been defined as the extent to which an individual tends to use the technology automatically [22]. A habit is often considered a reflective behavior or action taken without much consideration. With time, ideas, methods, judgments, and reactions stabilize. Such a behavioral model becomes partially fixed; thus, it is referred to as inertial. Habits are also reported to affect previous and future behavior [47]. Gefen [48] reported that the use of technology becomes a personal habit for users if the use of innovative technology is routine behavior. Habits strengthen a personal behavior through repeated stimulation and reaction. Limayem et al [22] noted that users who have used mHealth technologies for more than one year gain habits that involve using technologies. Thus, habits were expected to influence the infusion of mHealth technologies and are considered to significantly influence mHealth continuance.

Goodhue and Thompson [37] indicated that a better fit between task and technology characteristics improves performance in the infusion stage when technology has been used continuously. Therefore, task and technology characteristics should be considered while investigating technology performance. Task characteristics imply the inherent nature of tasks that users are expected to execute. In this study, task characteristics comprised time criticality, interdependence, and mobility. Time criticality is defined as urgency when accessing information through mHealth technology [33,49]. Interdependence is the degree to which completing tasks using mHealth technology requires interaction with other people [33,50]. Mobility is the extent to which a task can be performed in different locations using mHealth technology [33,49]. Technology characteristics are the specific features, functionality, or usability provided by specific technologies. Technology characteristics comprise availability, maturity, and portability. Availability is defined as the ability to access mHealth technologies when required [33,51]. Maturity is related to the existence of a level of system quality that is perceived as satisfactory and the perceived need for system improvement by users [33,43,52]. Portability is the degree of ease associated with transporting mHealth technologies [33,53]. As shown, 3 research hypotheses, including 8 subhypotheses, are proposed as follows:

- H7: The individual characteristics of HCPs significantly affect mHealth continuance.
  - H7a: Personal innovativeness significantly affects mHealth continuance.
  - H7b: Individual habits significantly affect mHealth continuance.
- H8: The technology characteristics of mHealth significantly affect mHealth continuance.
  - H8a: The availability of mHealth significantly affects mHealth continuance.
  - H8b: The portability of mHealth significantly affects mHealth continuance.
  - H8c: The maturity of mHealth significantly affects mHealth continuance.
- H9: The task characteristics of HCPs significantly affect mHealth continuance.
  - H9a: Task time criticality significantly affects mHealth continuance.
  - H9b: Task interdependence significantly affects mHealth continuance.
  - H9c: Task mobility significantly affects mHealth continuance.

### Instrument and Respondents

The questionnaire was designed in 2 stages. The first stage involved the establishment of measurement items. We collected results from literature reviews to obtain a comprehensive list of measurement items. All measures for each construct were obtained from existing validated instruments, and they were modified to ensure the appropriateness for mHealth. A total of 4 variables, namely confirmation, perceived usefulness, satisfaction, and mHealth continuance, which were derived from the ECM, were measured using 15 items adapted from Bhattacherjee [20], Mettler [25], Wright and Marvel [42], and Kuo et al [41]. The performance was measured using 6 items adapted from O’Connor et al [33] and Junglas et al [44]. Individual characteristics, including innovativeness and habits, were measured using 8 items adapted from Limayem et al [22], Chen et al [29], O’Connor et al [33], and Kuo et al [41]. Technology characteristics, including availability, portability, and maturity, were measured using 9 items adapted from Lin [17], O’Connor et al [33], and Gebauer et al [43]. Task characteristics, including time criticality, interdependence, and mobility, were measured using 9 items adapted from Hsiao and Chen [32], Lin [17], and O’Connor et al [33]. The detailed descriptions related to the survey questionnaire are provided in the Multimedia Appendix 1. The questionnaire items were preliminarily translated into Chinese, and 2 experts in bilingual education in health care and information management were invited to evaluate the content equivalence of the translations. The questionnaire comprised 2 major parts. The first part collected participants’ demographic data, including age, sex, education level, department, and experience in using mobile technologies and mHealth. The second part included measure items related to the factors influencing mHealth continuance and performance.

The second stage of questionnaire design involved the evaluation and selection of the measurement scale. A content validity index (CVI) was used to evaluate the questionnaire content according to a threshold value of 0.8 for item selection suggested by Petrick [54]. A total of 2 HCPs of mHealth and a professor of health informatics management were invited as experts to examine the content validity of the questionnaire. Among the initial questionnaire containing 47 items, except for 1 item, which was excluded as its CVI was less than .8, 46 items were retained as the CVI values were greater than .95 and the average CVI was .98, which indicated excellent expert validity. Furthermore, the semantics and wording of the questionnaire were revised according to experts’ suggestions. Finally, a 46-item questionnaire was obtained. Each item was measured using as 5-point Likert scale (1 for strongly disagree and 5 for strongly agree).

The respondents of this study were the HCPs of the target hospital with approximately 120 doctors and 500 nurses in southern Taiwan. Since 2009, the case hospital has developed and implemented mHealth systems, a combination of mobile ISs and medical devices, for satisfying HCPs’ needs of clinical patient care, particularly for providing more timely communication of HCPs and direct data input at source, reducing possible medical errors, and accessing up-to-date medical records. The mobile ISs can connect and access all required and integrated patient-related information from hospital ISs, including various developed systems (computerized physician order entry system, laboratory ISs, nursing ISs, pharmacy ISs, picture archiving and communication system, electronic medical records, patient referral system, and others) for supporting inpatient, outpatient, and emergency services in a hospital, through a secure wireless network infrastructure. The mobile ISs can be installed on various mobile devices, including a mobile nursing cart equipped with a Tablet PC (specifically for nurses), mobile medical cart equipped with a Tablet PC (specifically for physicians), mobile phones, and iPad for satisfying the mobile needs of HCPs, particularly in the inpatient and emergency services.

Since 2014, some health apps of the case hospital have been developed and installed on mobile phones and iPads for providing instant access to the results of medical examinations, tests, and reports and receiving immediate notifications from high-risk reminder systems for clinical laboratory critical value alerts; however, those apps only provide relatively specific and limited information for patient care because of the limitations of small screen size, less computation power, and data key-in problems of intelligent mobile devices. Therefore, HCPs in the case hospital prefer accessing full patient care information through mobile ISs installed on the mobile nursing cart, mobile medical cart, and tablet PC. HCPs who had at least one year of experience in mHealth apps and were active and voluntary users of mHealth, using mobile ISs through mobile devices in clinical practices, were requested to participate. After obtaining approval from the Institutional Review Board (IRB NO.105B-009), the questionnaires were distributed to qualified HCPs under the assistance of the nursing department and hospital administration department. The duration of data collection was from February 1 to March 1 in 2016.

## Results

### Descriptive Statistics

The survey was administered to 282 respondents, and 201 valid responses were returned, which indicated a response rate of 71.3% (201/282). Voluntary participation might explain the relatively high response rate. The demographic data (see Table 2) showed that most of the respondents (94.0% [189/201]) were female, 92.1% (185/201) were less than 40 years old (48.3% [97/201] and 43.8% [88/201] were aged <30 years and 30-40 years, respectively), and 73.1% (147/201) had a bachelor’s or master’s degree. Among the respondents, 94.0% (189/201) worked in the nursing department, whereas the others worked in the medical department. Moreover, 77.1% (155/201) of the participants had more than 1 year of experience in using mHealth, thus indicating the appropriateness of the selected respondents.

**Table 2:** Demographic data (n=201).

Measure or category		Statistics
**Age (years), n (%)**		
	<30	97 (48.3)
	31-40	88 (43.8)
	41-50	12 (6.0)
	51-60	4 (2.0)
**Gender, n (%)**		
	Male	12 (6.0)
	Female	189 (94.0)
**Education level, n (%)**		
	Junior college	54 (26.9)
	Bachelor	144 (71.6)
	Master (or higher)	3 (1.5)
**Department, n (%)**		
	Medical (Physicians)	12 (6.0)
	Nursing (Clinical nurses)	189 (94.0)
**Experience in using mobile technologies (years), n (%)**		
	1-3	146 (72.6)
	3-6	43 (21.4)
	6-9	7 (3.5)
	>9	5 (2.5)
**Experience in using mobile health (years), n (%)**		
	1	46 (22.9)
	1-5	145 (72.1)
	5-10	10 (5.0)

### Measurement Model

The collected data were analyzed using the partial least square (PLS) technique, which can offer extensive, scalable, and flexible causal-modeling capabilities [55], in the WarpPLS software (Version 5.0) because of its ease of use as well as its capability of performing all the modeling procedures reported in this study [56]. A 2-step approach of the PLS technique suggested by Chin [57] was used. The first step was to evaluate the measurement model, whereas the second step focused on evaluating the structural model. Several criteria are recommended for assessing the model-data fit when using WarpPLS 5.0, including average path coefficient (APC), the average R-squared (ARS), average adjusted R-squared (AARS), average block variance inflation factor (AVIF), average full collinearity variance inflation factor (AFVIF), Tenenhaus Goodness of Fit (GoF), and R-squared contribution ratio (RSCR) [56]. These model fit and quality indices are other advantages provided by WarpPLS 5.0 than other variance based structural equation modeling methods. In general, the addition of latent variables into a model will increase the value of ARS but decrease the value of APC. Both ARS and APC will increase simultaneously only when the addition of latent variables can improve the overall model predicative and explanatory quality [56]. The AARS, generally lower than ARS in a model, is used to correct improper increases in R-squared coefficients when predicators cannot improve the explanatory value in each latent variable [56]. The AVIF and AFVIF are used to evaluate the increase of collinearity of the model if new latent variables are added and that may overlap in meaning with existing latent variables [56]. The GoF is a measure for evaluating the model’s explanatory power, while RSCR is a measure evaluating the extent that a model is free from negative R-squared effects [56]. As demonstrated in Table 3, the results showed that all the model fit and quality indices are in the recommended range or have probability values less than .001. The APC is 0.237 for a *P*<.001, the ARS index is 0.529 for a *P*<.001, and the AARS index is 0.521 for a *P*<.001. All the values of APC, ARS, and AARS show a better fit than the recommended values. The AVIF is 2.246 and AFVIF is 2.324, representing there is no collinear problem found in the investigated model. The GoF is .649 which indicates a better fit than the large value of .36 [56]. In conclusion, the proposed model of mHealth is validated, representing good model fit and quality indices.

**Table 3:** Model fit and quality indices.

Quality indices	Statistics	Criteria (*P* value)	Result
Average path coefficient (APC)	0.237 (*P*<.001)	<.05	Fit
Average R-squared (ARS)	0.529 (*P*<.001)	<.05	Fit
Average adjusted R-squared (AARS)	0.521 (*P*<.001)	<.05	Fit
Average block variance inflation factor (AVIF)	2.246	Acceptable if ≤5, ideally ≤3.3	Fit
Average full collinearity VIF (AFVIF)	2.324	Acceptable if ≤5.0, ideally ≤3.3	Fit
Tenenhaus Goodness of Fit (GoF)	0.649	Small ≥.1, medium ≥.25, large ≥.36	Fit
R-squared contribution ratio (RSCR)	0.989	Acceptable if ≥ .9, ideally=1.0	Fit

We further evaluated the psychometric properties of the instrument regarding reliability, convergent validity, and discriminate validity. According to the method used by Hair et al [58], Cronbach alpha and the composite reliability (CR) of each construct was used to test reliability and internal consistency. Table 4 showed that the values of Cronbach alpha and CR of all the constructs were higher than the recommended value (0.7) [56,58], thus exhibiting acceptable reliability and internal consistency. The validity of the measures was tested using convergent and discriminant validity. Fornell and Larcker [59] recommended that the average variance extracted (AVE) value should exceed .5 and each square correlation, which indicated adequate convergent validity and discriminant validity. As shown in Table 4, the AVE values of all constructs were between .686 and .898, which are greater than the recommended value (.5), thus demonstrating an excellent convergent validity. Furthermore, all the square roots of AVE were higher than any other correlation among the latent variables, thus indicating an adequate discriminant validity.

### Structural Model

**Figure 2:**
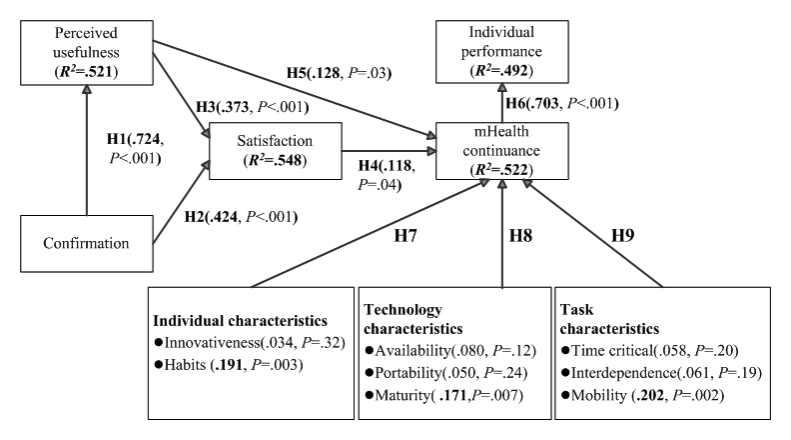
Results of the model validity. H1: The confirmation of mHealth systems significantly affects perceived usefulness; H2: The confirmation of mHealth systems significantly affects user satisfaction; H3: The perceived usefulness of mHealth systems significantly affects user satisfaction; H4: User satisfaction with mHealth systems significantly affects mHealth continuance; H5: The perceived usefulness of mHealth systems significantly affects mHealth continuance; H6: The continuance of mHealth significantly affects individual performance; H7: The individual characteristics of HCPs significantly affect mHealth continuance; H8: The technology characteristics of mHealth significantly affect mHealth continuance; H9: The task characteristics of HCPs significantly affect mHealth continuance; mHealth: mobile health.

The structural research model was analyzed using WarpPLS 5.0 and the bootstrap resampling method [56]. Testing of the structural model was mainly on the basis of the path coefficient and *R*^2^ value. Path coefficients represent the strength and direction of the relation among variables to test their significance, whereas *R*^2^ values indicate the percentage to which external variables can explain the variability of internal variables and indicate the predictive power of the model. As shown in Figure 2, nine hypotheses (H1, H2, H3, H4, H5, H6, H7b, H8c, and H9c) were confirmed, whereas the remaining hypotheses (H7a, H8a, H8b, H9a, and H9b) were not significantly supported by this study. The results revealed that mHealth continuance (*R*^2^=0.522) is mainly affected by perceived usefulness (beta=.128; *P*=.03), maturity (beta=.171; *P*=.007), habits (beta=.191; *P*=.003), task mobility (beta=.202; *P*=.002), and user satisfaction (beta=.118; *P*=.04), whereas individual performance (*R*^2^=0.492) is affected by mHealth continuance use (beta=.703; *P*<.001). In addition, user satisfaction (*R*^2^=0.548) is affected by confirmation (beta=.424; *P*<.001) and perceived usefulness (beta=.373; *P*<.001). Confirmation (beta=.724; *P*<.001) significantly affected perceived usefulness (*R*^2^=0.521) and user satisfaction (*R*^2^=0.548).

**Table 4:** Results of the reliability and validity of the research model.

Construct	CO^a^	PU^b^	SAT^c^	INN^d^	HAB^e^	AVA^f^	TC^g^	INT^h^	MC^i^	PER^j^	MOB^k^	PORT^l^	MAT^m^	AVE^n^ (>.5)	CR^o^ (>.7)	Cronbach alpha (>.7)
CO	0.898	—^p^	—	—	—	—	—	—	—	—	—	—	—	0.806	0.943	.919
PU	0.718	0.869	—	—	—	—	—	—	—	—	—	—	—	0.755	0.939	.918
SAT	0.690	0.657	0.894	—	—	—	—	—	—	—	—	—	—	0.798	0.922	.874
INN	0.265	0.338	0.309	0.867	—	—	—	—	—	—	—	—	—	0.751	0.924	.889
HAB	0.586	0.500	0.502	0.320	0.910	—	—	—	—	—	—	—	—	0.829	0.951	.931
AVA	0.509	0.522	0.584	0.306	0.504	0.855	—	—	—	—	—	—	—	0.730	0.890	.815
TC	0.450	0.511	0.475	0.361	0.387	0.513	0.887	—	—	—	—	—	—	0.787	0.917	.864
INT	0.344	0.433	0.358	0.332	0.280	0.517	0.707	0.918	—	—	—	—	—	0.843	0.942	.906
MC	0.528	0.564	0.534	0.320	0.534	0.586	0.529	0.503	0.914	—	—	—	—	0.836	0.939	.901
PER	0.628	0.664	0.638	0.406	0.535	0.610	0.541	0.521	0.695	0.895	—	—	—	0.802	0.960	.950
MOB	0.302	0.406	0.286	0.245	0.326	0.410	0.476	0.582	0.463	0.444	0.947	—	—	0.897	0.946	.886
PORT	0.352	0.355	0.403	0.200	0.297	0.574	0.452	0.434	0.410	0.462	0.271	0.828	—	0.686	0.868	.771
MAT	0.440	0.477	0.506	0.249	0.340	0.653	0.547	0.517	0.518	0.606	0.337	0.621	0.899	0.809	0.927	.881

^a^CO: confirmation.

^b^PU: perceived usefulness.

^c^SAT: satisfaction.

^d^INN: innovativeness.

^e^HAB: habits.

^f^AVA: availability.

^g^TC: time critical.

^h^INT: interdependence.

^i^MC: mobile health continuance.

^j^PER: performance.

^k^MOB: mobility.

^l^PORT: portability.

^m^MAT: maturity.

^n^AVE: average variance extracted.

^o^CR: composite reliability.

^p^The omitted correlation coefficients between constructs in the upper diagonal matrix are equal to the values in lower diagonal matrix.

## Discussion

### Key Factors Affecting Perceived Usefulness and User Satisfaction

Consistent with previous ECM-related studies [26,27], confirmation (beta=.724; *P*<.001) is a factor that significantly affected perceived usefulness (by the HCPs) and user satisfaction. Both confirmation (beta=.424; *P*<.001) and perceived usefulness (beta=.373; *P*<.001) were significant predicators of user satisfaction with the mHealth systems. In the context of eHealth, confirmation accounted for 52.1% variance of perceived usefulness, whereas both confirmation and perceived usefulness accounted for 54.8% variance of satisfaction. Confirmation refers to users’ perception of the congruence between the expectation of mHealth use and its actual performance [20], whereas perceived usefulness is the perception of users’ regarding the expected benefits of mHealth use [20,41]. This implied that the expectations of the HCPs from the mHealth systems were confirmed through performance after implementation. The participants expected that mHealth use would positively affect the quality of clinical care, the effectiveness of medical teams for simultaneous processing of patient information, and patient care management. Understanding the expectations of the HCPs before system development and evaluating their responses after implementation of the mHealth system can increase the benefits of using the mHealth system in clinical care. Thus, continuous evaluation of whether the clinical care functions provided by the mHealth system meet the expectations of users is crucial. As the HCPs continuously use the system over a long time and become more familiar with it, they may have new requirements for further improvement of the system. Dynamic changes in functional requirements should be considered by managers and system developers for ensuring user satisfaction. After mHealth is infused and integrated into the daily operations and clinical care practices of HCPs, the HCPs should compare their preadoption expectations and actual performance as well as perceived usefulness of the mHealth systems, thus improving the quality of clinical care and efficiency of care management. Such confirmations and perceived usefulness of mHealth are helpful in improving user satisfaction.

This study showed that perceived usefulness (beta=.128; *P*=.03), user satisfaction (beta=.118; *P*=.04), technology maturity (beta=.171; *P*=.007), individual habits (beta=.191; *P*=.003), and task mobility (beta=.202; *P*=.002) exert significantly positive effects on mHealth continuance, which accounted for 52.2% of the total explained variance. Among the identified factors that affected mHealth continuance, task mobility, individual habits, and technology maturity have more significant direct effects on mHealth continuance than the factors (perceived usefulness and user satisfaction) derived from the ECM. Consistent with previous ECM-related studies [26,27], this study confirmed that perceived usefulness and user satisfaction were key predicators of mHealth continuance. In addition, the inclusion of characteristics of individuals, technology, and tasks facilitated the extension of the original ECM for understanding the factors influencing mHealth continuance. To increase the HCPs’ intention toward mHealth continuance, paying attention to the characteristics related to task mobility, user habits, technology maturity, and user perceptions related to perceived usefulness, and user satisfaction is necessary.

This study made an empirical validation on the framework of mHealth infusion proposed by O’Connor et al; however, only mobility, habit, and maturity were found to be salient predicators in mHealth infusion. Previous studies have highlighted that mobility is the primary reason for the applications of technological innovation in hospitals [14-16]. Zhang et al [49] reported that mHealth technologies offer the staff freedom to interact with and use technological tools irrespective of time and location. This study showed that mobility is the most crucial factor influencing mHealth continuance. Consistent with the results of a study by Limayem et al [22], this study revealed that habits play a major role in mHealth continuance. Previous studies have indicated that maturity is related to the existence of a level of system quality that is perceived as satisfactory and the perceived need for system improvement by the user [33,43,52]. O’Connor et al [33] argued that poor graphical user interface design and unsatisfactory process design of mobile systems result in unnecessary medical errors. When users (the HCPs) perceive poor quality of the mHealth systems, they are less likely to use the mHealth systems. Our study revealed that mHealth maturity was critical to its continuance. Therefore, it increased the intention of HCPs toward mHealth continuance by focusing on the design and implementation issues of the mHealth applications to satisfy actual users’ needs. This study indicated that when the mHealth applications provide high quality (of mHealth system) and superior support for the HCPs’ needs, the users (HCPs) had a relatively high intention toward mHealth continuance. As stated, we suggested the evaluation of task mobility, technology maturity, and individual habits and the provision of better support related to the fit among the aforementioned factors while introducing mHealth applications as those factors are salient predicators for mHealth continuance by the HCPs.

Furthermore, the perceived usefulness and user satisfaction of mHealth systems have been considered as critical factors affecting technology continuance in ECM-related studies [26,27], and they have been reported to exert significantly positive effects on mHealth continuance from the HCPs’ perspective in this study. We should provide sufficient incentives and resources to improve perceived usefulness (by the HCPs) and user satisfaction after implementation of the system or in the infusion stage. Contrary to previous studies [33,34,49,50,53], we found that some factors in the characteristics of individuals (innovativeness), technology (availability and portability), and task (timeliness and interdependence) did not significantly affect mHealth continuance in this study. A possible explanation may be that aforementioned factors were not salient predicators in the mHealth context, particularly after implementation and in the infusion stage of the mHealth systems from the HCPs’ perspective in Taiwan. In addition, those nonsignificant factors are mainly derived from the conceptual framework of mHealth infusion proposed by O’Connor et al [33] and they may obtain mixed results through empirical studies because of the difference of research contexts, user groups, and application systems. It is acceptable that some factors investigated in this study were insignificant in the mHealth applications of the case hospital in Taiwan from the HCPs’ perspective. For example, consumer’ personal innovativeness is a significant factor of mHealth assimilation in Rai et al [34]; however, we found personal innovativeness is insignificant in the health care context from the HCPs’ point of view.

### Key Factors Affecting Individual Performance

The results indicated that mHealth continuance (beta=.703; *P*<.001) exerted significantly positive effects on individual performance, thus explaining 49.2% variance in individual performance. O’Connor et al [33] found that the individual performance of HCPs was influenced by continued mHealth use in the infusion stage, which also significantly affected individual performance in system use. As expected, consistent with O’Connor et al [33] and Goodhue and Thompson [37], this study highlighted that mHealth continuance positively affected individual performance. If the HCPs intend to incorporate the mHealth systems into routine practices in the postimplementation or infusion stage, mHealth systems can enhance their individual performance, including improving the information exchange within a medical team and task identity in clinical care, increasing the efficiency of patient care, enhancing the quality of clinical patient care, and improving communication between health care personnel and patients or their families. System adoption in an organization is not always voluntary; sometimes it is because of work requirements or the necessity of IS for work completion. The ultimate goal of the system development process, including initial conception, implementation, adoption, and the following acceptance and continued use of mHealth, is to improve individual performance and satisfy clinical work demands. Therefore, we need to pay attention to system functions and demands of mHealth that require further improvement; thus, users will become more familiar and comfortable with mHealth. This is helpful to improve user work performance.

Furthermore, in this study, we evaluated the individual performance of the HCPs derived from mHealth continuance by using 6 items (Table 5). The results showed that the average score of each item ranged between 3.83 and 4.10, which indicated a positive evaluation by HCPs on mHealth continuance. According to the results in a descending ranking of the average score of each item, the HCPs perceived that the use of an eHealth system improved information exchange with the health care team (mean 4.10, SD 0.60), facilitated communication with patients and their families (mean 4.10, SD 0.60), provided efficient patient care (mean 3.94, SD 0.60), enhanced the quality of patient care (mean 3.91, SD 0.63), improved professional image (mean 3.86, SD 0.63), and facilitated task completion (mean 3.83, SD 0.62). This implied that improving information exchange with health care teams, facilitation of communication with patients and their families, and providing efficient patient care were the top 3 measures of performance of the mHealth systems identified by the HCPs.

**Table 5:** Individual performance derived from mobile health continuance.

Items	Mean (SD)
Using mHealth^a^ can effectively improve information exchange between me and the health care team	4.10 (0.60)
Using mHealth can effectively facilitate my communication with patients and their families	4.10 (0.60)
Using mHealth allows me to provide efficient patient care	3.94 (0.60)
Using mHealth enhances the quality of patient care	3.91 (0.63)
Using mHealth improves my professional image	3.86 (0.63)
Using mHealth facilitates my work completeness	3.83 (0.62)
Average score	3.96 (0.61)

^a^mHealth: mobile health.

### Conclusions

The key building block for sHealth care is mHealth, and the appropriate use of mHealth may result in major advances in expanding health care coverage, improving decision making, managing chronic conditions, and providing suitable health care during emergencies [9]. However, previous studies have indicated that mHealth is in its early stages of development [1]. Moreover, neither does current mHealth research adequately evaluate mHealth interventions nor does it provide sufficient evidence on the effects of mHealth on health [10]. Thus, appropriate evaluation, specifically after the implementation of mHealth systems and the use of the systems in daily health care practices, from the users’ perspectives is critical. This study proposed an innovative extended model by integrating the ECM and characteristics of individuals, technology, and tasks to investigate critical factors affecting the continuance of mHealth and the performance of mHealth from the HCPs’ perspective and assessing the infusion of the mHealth systems in clinical practices.

The results revealed that mHealth continuance was mainly affected by perceived usefulness, technology maturity, individual habits, task mobility, and user satisfaction, whereas individual performance was influenced by mHealth continuance. User satisfaction was affected by confirmation and perceived usefulness of mHealth, whereas perceived usefulness was affected by confirmation. This study showed that the ECM remained valid in the mHealth context from the HCPs’ perspective. Among the identified factors that influenced mHealth continuance in this study, task mobility, individual habits, and technology maturity affected mHealth continuance more significantly than the factors (perceived usefulness and user satisfaction) derived from the ECM. To increase the intention of health professionals toward mHealth continuance, characteristics related to task mobility, user habits, and technology maturity and users’ perceptions related to perceived usefulness and user satisfaction must be given attention.

We found that the users’ intention toward mHealth continuance increased when the focus was on the design and implementation issues of the mHealth applications to satisfy the actual needs of users. This implied that if mHealth applications provided high quality of system and satisfactory support to meet the needs of the HCPs, the users will have a relatively high intention toward mHealth continuance. We further suggested the evaluation of task mobility, technology maturity, and individual habits and provision of satisfactory support related to the fit between the aforementioned factors while introducing mHealth applications. Consistent with the results of previous ECM-related studies [26,27], we found that perceived usefulness and user satisfaction were the key factors affecting mHealth continuance from the HCPs’ perspective. This study reported that confirmation played a key role in affecting perceived usefulness and user satisfaction. This indicated that the perceived usefulness and user satisfaction were effectively improved by minimizing the gaps between user expectations of mHealth use and its actual performance. We suggested the minimization of the gaps between user expectations of mHealth use and its actual performance by providing sufficient incentives and resources to improve the perceived usefulness and user satisfaction after implementation or in the infusion stage.

This study has made theoretical and practical contributions to the evaluation of mHealth systems. First, the study proposed an innovative integration model that extended the ECM with antecedents of IS infusion (including the characteristics of individuals, technology, and tasks) to identify the critical factors influencing mHealth continuance and performance from the perspective of HCPs. The extended ECM provided a comprehensive research model for investigating mHealth continuance or IS continuance. Second, the inclusion of characteristics of individual, technology, and task not only provided a reasonable framework but also highlighted that other studies can incorporate various critical factors depending on research contexts and situations. Third, the identified critical and salient factors that affected mHealth continuance and performance can be used as assessment tools by hospitals that have implemented mHealth to facilitate mHealth use and infusion. (4) The results can also help health care institutions that intend to introduce or develop mHealth applications in identifying critical issues and effectively allocating limited resources to mHealth systems.

We suggest focus areas for additional research and future studies on this topic. First, scholars can use the research model derived in this study, apply it to various research contexts, and compare the findings. Second, others can conduct an in-depth case study with the findings obtained from this study. To expand the research scope at the IS infusion stage, future studies should pay attention to the investigated factors (personal innovativeness, availability, portability, timeliness, and interdependence) that were insignificant factors in this study. This is reasonable as those insignificant factors may have different (mixed) results because of the difference of research contexts, user groups, and application systems as mentioned in a summary of technology acceptance model studies [60].

This study has several limitations. First, this study was conducted only at a regional hospital in Taiwan; thus, the findings obtained from this research may not be immediately transferrable to other countries with different participant demographics and cultures. Second, a cross-sectional survey design was used for this study; thus, the inherent limitations of the survey methodology were inevitable. Furthermore, this study sample comprised voluntary participants. However, as the survey approach is commonly used in the field, the use of this method may not have adversely affected the results.

## Appendix

Multimedia Appendix 1 Questionnaire for mobile health continuance and performance.

## Abbreviations

AARS: average adjusted R-squared
AFVIF: average full collinearity variance inflation factor
APC: average path coefficient
ARS: average R-squared
AVE: average variance extracted
AVIF: average block variance inflation factor
CR: composite reliability
CVI: content validity index
ECM: expectation-confirmation model
eHealth: electronic health
GoF: Goodness of Fit
HCP: health care professional
HIS: hospital information system
ICT: information communication technology
IS: information system
IT: information technology
mHealth: mobile health
PC: personal computer
PLS: partial least square
RSCR: R-squared contribution ratio
sHealth: smart health
TTF: task-technology fit
