# Supplementary material for: Understanding Determinants of Health Care Professionals’ Perspectives on Mobile Health Continuance and Performance
Source: JMIR Med Inform. 2019 Mar 18;7(1):e12350. doi: 10.2196/12350 (PMC6441862; doi:10.2196/12350)
Supplement: Multimedia Appendix 1 [file medinform_v7i1e12350_app1.pdf]

Appendix

Questionnaire for mHealth continuance and performance.

CO1: Using the MH is better for improving the quality of clinical care than I expected.

CO2: Using the MH is better for improving the effectiveness of medical team to process patient information at the same time than I expected.

CO3: Using the MH is better for improving patient care management than I expected

CO4*: Using the MH is better for effective implementation of the clinical Practices guidelines of patients care than I expected.

PU1: Using the MH in my job will increase my productivity.

PU2: Using the MH in my job will make me more effective.

PU3: Using the MH is more effective in assisting in the collection and analysis of patient data

PU4: Using the MH will reduce the manual handling of materials.

PU5: Using the MH will improve communication with the medical team.

SAT1: My experience with using MH is quite close to my ideal MHS.

SAT2: My overall experience of MH use was absolutely delighted.

SAT3: Overall, I am satisfied with the use of MH.

MC1: I intend to continue using MH rather than discontinue its use.

MC2: My intentions are to continue using MH than use any alternative means.

MC3: I will use MH regularly in the future.

INN1: Others come to me for advice on new technology.

INN2: I am among the first in my circle of friends to acquire new technology.

INN3: I can figure out new high-tech products and services without any help.

INN4: I can keep up with the latest technological development that I am interested in.

HAB1: The use of the MH has become a routine practice when providing healthcare services

HAB2: The use MH to perform clinical practice is my preferred way of performing tasks.

HAB3: Using the MH has become automatic to me.

HAB4: The use of MH has become a habit for me.

AVA1: I have no difficulty in finding out a MH to use when required.

AVA2: When providing healthcare services, availability of MH is not a problem.

AVA3: There are sufficient amount of MH for me to use in the department in which I am predominantly located.

PORT1: Mobile medical device has the convenience of moving around.

PORT2: MHS can provide services anywhere in the hospital.

PORT3: The mobile medical battery's sustainability continues to meet my clinical work needs.

MAT1: The function of MH can meet the needs of my clinical work.

MAT2: The MH has full functions for my clinical work.

MAT3: The function of MH is enough for me to complete routine tasks.

TC1: I often need to send various information to other medical personnel immediately when I am doing clinical practices.

TC2: I use MH to access patient information in emergency situations.

TC3: I use MH to help me make clinical decisions in urgent situations.

INT1: I often need to immediately control the patient in critical condition with other medical professionals.

INT2: I need to work with professionals to solve the clinical problems of the patient.

INT3: My clinical work is often cross-departmental.

MOB1: I often need to move in different locations when I do my clinical work.

MOB2: I need to constantly move in my workplace according to various situations when I am doing clinical work.

MOB3: The freedom to choose clinical work in a fixed working location is not high.

PER1: Using MH can improve the message exchanges between healthcare professionals.

PER2: Using MH can increase efficiency of patient care activities.

PER3: Using MH can improve the quality of care.

PER4: Using MH can increase the professional image of healthcare personnel.

PER5: Using MH can improve the overall performance in clinical practices.

PER6: Using MH can facilitate communication with patients.

Note: 1. CO: confirmation; PU: perceived usefulness; SAT: satisfaction; INN: innovativeness; HAB: habits; TC: time critical; MOB: mobility; AVA: availability; PORT: portability; MAT: maturity; INT: interdependence; MC: mHealth continuance; PER: performance; MH: mobile health; MHS: mobile health systems

2. * indicates the excluded item for further analysis
